# Acute renal infarction resulting from fibromuscular dysplasia: a case report

**DOI:** 10.1186/s13256-016-0895-6

**Published:** 2016-05-10

**Authors:** Harri Juhani Saarinen, Ari Palomäki

**Affiliations:** 1 Department of Cardiology, Kanta-Häme Central Hospital, Ahvenistontie 20, 13530 Hämeenlinna, Finland; 2 Department of Emergency Medicine, Kanta-Häme Central Hospital, Ahvenistontie 20, 13530 Hämeenlinna, Finland; 3 Medical School, University of Tampere, 33014 Tampere, Finland

**Keywords:** Renal infarction, Renal artery, Kidney, Thrombosis, Abdominal pain, Fibromuscular dysplasia

## Abstract

**Background:**

Acute abdominal pain is one of the most frequent complaints evaluated at emergency departments. Approximately 25 % of abdominal pain patients discharged from emergency departments are diagnosed with undifferentiated abdominal pain. One possible reason for acute abdominal pain is renal infarction. Diagnosis is difficult and often late.

**Case presentation:**

A white, 33-year-old, previously healthy Finnish man came to our emergency department because of acute abdominal pain. After evaluation and follow-up he was discharged the next day with a diagnosis of undifferentiated abdominal pain. He returned a day later and was diagnosed with renal infarction. Appropriate therapy was initiated in the nephrology ward. Further tests confirmed a diagnosis of renal infarction as a result of fibromuscular dysplasia. He recovered well and was discharged on the tenth day of hospitalization. His renal function was normal.

**Conclusions:**

Renal infarction is rare and should be considered if a patient with intense flank pain has no sign of urolithiasis or pyelonephritis. Contrast-enhanced computer tomography and assay of lactate dehydrogenase are recommended. The optimal treatment is still uncertain. Every patient discharged with undifferentiated abdominal pain should be given clear instructions as to when it is necessary to return to the emergency department.

## Background

Acute abdominal pain is one of the most frequent presenting complaints evaluated at emergency departments (EDs) and it represents 5–10 % of ED visits [1, 2]. Despite advanced diagnostic modalities, approximately 25 % of abdominal pain patients discharged from EDs are diagnosed with undifferentiated abdominal pain [1]. Diagnosis can sometimes be difficult, and thus diagnostic laparotomy is commonly carried out. Vascular emergencies might arise as one of the most difficult diagnostic problems [3]. The annual incidence of acute renal infarction in patients referred to EDs has been reported to be 0.007 % in retrospective studies [4, 5]. Presenting symptoms of renal infarction are not unique and the time gap between the onset of symptoms to diagnosis is often nearly 2 days [6, 7]. Helical computed tomography (CT) scanning without contrast is the gold standard for the more common kidney and ureteral stones, thus being often the first imaging test as regards flank pain. If there are no signs of urolithiasis, a contrast-enhanced CT scan should be carried out to assess the possible occurrence of renal infarction. The classic finding in a case of renal infarction is a wedge-shaped perfusion defect.

## Case presentation

A white, 33-year-old, previously healthy Finnish man came to the ED of our hospital in Finland because of intense abdominal pain. He was a nonsmoker and had no history of alcohol abuse. Our patient had had no previous medication and he denied any acute drug usage. He had undergone appendectomy 15 years previously. He had no history of traffic accident-related or other abdominal trauma. The acute pain was located on the left side of his abdomen, running down to the left inguinal area. He rated the pain as maximal, numerically 10 on a 1–10 scale. On clinical examination his abdomen was soft on palpation with no abdominal guarding, but our patient was struggling with pain. Peripheral pulses were palpable and there was no tenderness on palpation of the kidneys. The symptoms supported a diagnosis of urolithiasis.

**Fig. 1:**
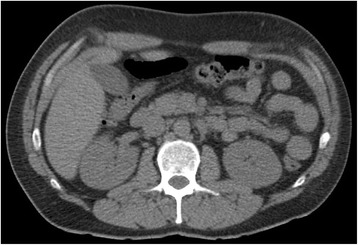
A helical computed tomography scan performed during the first emergency department visit showing no sign of urolithiasis. The kidneys are normal

**Fig. 2:**
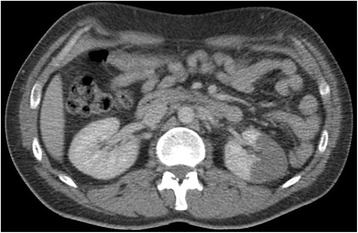
A contrast-enhanced abdominal computed tomography scan performed a day after the first visit reveals a renal infarction of the left kidney

Our patient was treated with intravenous oxycodone and his pain was relieved. A helical CT scan showed no sign of urolithiasis and his kidneys were normal (Fig. 1). Laboratory analysis showed an elevated white blood cell (WBC) count of 13.4 × 10^9^/L. However, his serum concentration of C-reactive protein (CRP) was normal, as were other laboratory test results (Table 1). Next morning, our patient was feeling well and had no abdominal pain. His WBC count had decreased to 10.0 × 10^9^/L. Otherwise the results were still normal. Our patient was discharged and advised to return should the pain reoccur. He returned to the ED the next evening because of rapid-onset intense abdominal pain located in the lower left quadrant of his abdomen, radiating to the left testicle. A urologist was consulted because of the possibility of testicular torsion, but our patient's clinical findings did not support this. The provisional diagnosis was still urolithiasis. Owing to the intense pain, explorative surgery was planned in order to assess the testicles and kidneys. Before any definite decision, contrast-enhanced abdominal CT was carried out. This revealed renal infarction of the left kidney (Fig. 2). The renal artery was open. After consultation, our patient was admitted to the nephrology ward.

**Table 1:** Laboratory results at the first emergency department visit

	Day 1	Day 2	Reference range
Parameter			
Blood hemoglobin (g/L)	133	121	134–167
Blood hematocrit (%)	40	36	39–50
Blood white cell count (x 10^9^/L)	13.4	10.0	3.4–8.2
Plasma sodium (mmol/L)	138	140	135–146
Plasma potassium (mmol/L)	3.50	3.50	3.3–3.5
Plasma creatinine (μmol/L)	61	79	60–100
Plasma C-reactive protein (mg/L)	<1	<1	0–10
Plasma alanine aminotransferase (U/L)	N/A	17	10–70
Plasma aspartate aminotransferase (U/L)	N/A	26	15–45
Plasma alkaline phosphatase (U/L)	N/A	50	35–105
Plasma amylase (U/L)	N/A	36	25–120
Urine glucose	Negative	N/A	Negative
Urine ketones	Negative	N/A	Negative
Urine blood	Negative	N/A	Negative
Urine albumin	Negative	N/A	Negative
Urine nitrite	Negative	N/A	Negative
Urine white cell screen	Negative	N/A	Negative

Our patient was started on enoxaparin, 100 mg twice daily, and examined for the possible source of emboli or a thrombophilic state. An electrocardiogram (ECG), transthoracic echocardiography and 48-hour continuous ECG monitoring did not indicate cardiac disease, arrhythmia or any other reason for suspected emboli. The results of laboratory tests for anticardiolipin antibodies and other markers of thrombophilic states including antithrombin III, protein C, protein S, lupus anticoagulant, beta 2 glycoprotein 1 antibodies, factor V Leiden, and factor II prothrombin were negative. There were no systemic clinical symptoms in our patient’s medical history such as fatigue, weight loss, arthralgia, hemoptysis, epistaxis, or persistent nasal crusting that would have suggested possible vasculitis. No palpable purpura or other skin manifestations were found either. Neither were there any abnormalities in the usual laboratory tests for identifying vasculitis such as erythrocyte sedimentation rate, antinuclear antibodies, antineutrophil cytoplasmic antibodies against either protease 3 or myeloperoxidase, serum complement levels C3 and C4, and antiglomerular basement membrane antibodies. A biopsy examination of the involved tissue was not feasible. There was a typical increase of lactate dehydrogenase (LDH). His intense abdominal pain was treated with intravenous oxycodone delivered via a patient-controlled analgesia pump, and epidural bupivacaine anesthesia. Anti-factor Xa levels were measured to adjust the enoxaparin dosage and enoxaparin was paused during removal of the epidural catheter.

**Fig. 3:**
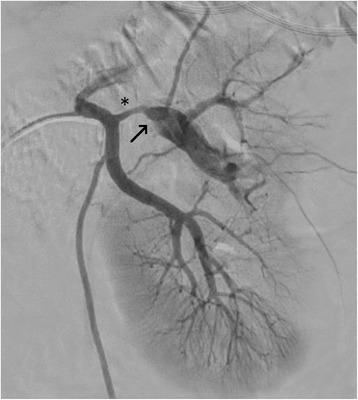
An invasive angiography of the left renal artery performed on the fifth day of hospitalization. The stenotic part is marked with an *asterisk*, followed by a dilated aneurysmatic part marked with an *arrow*

On the seventh day of hospitalization, our patient had a fever of 38.4 °C, an elevated WBC count and his CRP level had increased to 301 mg/L (Table 2). There appeared to be no focus of infection, but intravenous ceftriaxone was started because of possible secondary infection of the affected tissue. Invasive angiography of the left renal artery was performed on the fifth day of hospitalization. At first, the performing radiologist interpreted the finding as 10-mm-long stenosis of a segmental branch of the inferior renal artery, followed by 20 mm of poststenotic dilatation and obvious thrombosis (Fig. 3). The diameter of the stenotic part was approximately 1.7 mm. Our patient’s kidney function remained stable and normal, so invasive evaluation of a potential pressure gradient (which might have been difficult) was not found necessary at that time. Another theory arose when the findings were reevaluated at the next radiology meeting: the segmental branch of the inferior renal artery was evaluated as being aneurysmatic, and it was thought to have been a possible source of emboli.

**Table 2:** Selected laboratory data during the 10-day hospitalization period and follow-up visit 26 months after the patient was discharged

	At presentation	Peak	Discharged	Latest follow-up	Reference range
Parameter					
Blood white cell count (x 10^9^/L)	11.7	19.7	11.2	6.8	3.4–8.2
Plasma creatinine (μmol/L)	66	88	88	88	60–100
Plasma C-reactive protein (mg/L)	3	301	80	<1	0–10
Plasma lactate dehydrogenase (U/L)	N/A	659	343	194	105–205

Our patient was discharged on the tenth day of hospitalization. He was feeling well and the fever had subsided. Serum levels of CRP had decreased to 80 mg/L. Oral antibiotics (cephalexin), proton pump inhibitor, and 100 mg of enoxaparin once a day were prescribed for 1 week. Diuresis was normal. There was a slight rise in creatinine levels but his glomerular filtration rate (GFR) was normal (100 mL/min/1.73 m^2^). His clinical status was normal, with blood pressure (BP) of 136/80 mmHg.

Magnetic resonance angiography (MRA) was carried out 15 months after the infarction to check for possible progression of changes in the left renal artery. There were slight changes of caliber in the arteries of his left kidney. Similar changes were also noticeable in the common hepatic artery and superior mesenteric artery. The arteries of his right kidney were normal. The findings were evaluated as being a consequence of fibromuscular dysplasia (FMD) and our patient was started on acetylsalicylic acid (ASA), 100 mg once a day. A carotid ultrasound was performed and there were slight irregularities in the wall of his right common carotid artery, a finding which was suggestive of FMD. Doppler findings were normal and the carotid arteries were otherwise normal. A follow-up visit took place a year after the MRA examination. His BP measured by a nurse was 149/79 and his GFR was still normal. Our patient was instructed to perform home blood pressure monitoring and he later reported that his BP was 130–140/70–80. There appeared to be no problems during follow-up and our patient continued in his normal work.

## Discussion

Renal infarction is a rare condition with four identifiable groups: renal infarction with thromboemboli originating from the heart or aorta, renal infarction associated with renal artery injury (including renal artery dissection, FMD and Ehlers-Danlos syndrome with thrombotic aneurysms of the renal artery), renal infarction associated with hypercoagulability disorders, and idiopathic renal infarction. In the largest published series (94 patients with acute renal infarction), the idiopathic group represented 29 % [8]. Less common causes of renal infarction include renal artery occlusion following endovascular intervention [9] and cocaine use [10]. The symptoms of renal infarction are similar to those of many other causes of abdominal pain. An increased level of LDH is a typical finding as a common marker of cell necrosis [5, 11]. In differential diagnosis renal colic and acute pyelonephritis must be excluded. Neither of these conditions is associated with elevated LDH levels and in pyelonephritis the urine sample typically reveals pyuria, which is not typical in cases of renal infarction.

Our patient was first diagnosed with abdominal pain of an undetermined nature. Renal infarction was found later when he returned to the ED. Our patient had typical symptoms – an increased LDH level and a classic CT finding. The possibility of arterial disease as the primary reason for renal infarction was also proposed after the first radiologist interpreted the angiography finding as stenosis of the renal artery followed by a poststenotic dilatation and obvious thrombosis. In that case, rupture of the atherosclerotic plaque could be followed by the formation of local thrombosis and infarction, as in myocardial infarction (local thrombosis in a coronary artery) or cerebral infarction (thromboembolism from the carotid artery, for example, or local thrombosis) [12]. Unlike our case, atherosclerotic renal disease is known to be more common in patients aged 45 years or more [13].

Another theory was that renal infarction might have been a consequence of FMD, which is typically a finding among patients aged less than 50. FMD of renal arteries presents usually with hypertension, but cases of renal infarction have been reported [14]. Sometimes it progresses quickly and may lead to renal infarction because of thrombosis of the poststenotic dilatation of the renal artery. It has been stated that “FMD can easily be differentiated from atherosclerosis, in that it occurs in the middle or distal portions of the artery in younger patients without significant cardiovascular risk, whereas atherosclerosis occurs at the origin or proximal portion of the artery in older patients with cardiovascular risk factors” [15]. The young age of our patient was typical of FMD, but the stenosis was in a proximal part of a branch of the renal artery, not a distal part. Statin therapy and ASA were not initiated for our patient at discharge, since atherosclerosis was not supported by the age of our patient or the type of findings. Magnetic resonance angiography confirmed the finding to be a consequence of FMD and the renal infarction was classified as infarction associated with renal artery injury. The second most common site of involvement in FMD is carotid artery. In a recent registry study 73.3 % of adult patients in the United States Registry for FMD had extracranial carotid vessel involvement [16].

There are no comparative studies as regards the treatment of renal infarction. Reported approaches include anticoagulation, endovascular therapy, and open surgery. Only patients diagnosed very early may benefit from local low-dose thrombolysis [17]. Primary surgical therapy is not encouraged, with the possible exception of a trauma patient with other indications for surgery. Since the diagnosis is often delayed there is usually not much to be done about the infarction. The main focus is on preventing future ischemic events and treating the possible secondary problems related to the infarction. It is typical to treat patients with anticoagulation therapy, since many of them have a clear indication for it, for example, atrial fibrillation [4, 5, 8, 11]. There is no consensus of opinion on the duration of anticoagulation for patients with renal infarction and there are no reports comparing outcomes with those among untreated patients.

## Conclusions

Abdominal pain is a challenging problem in EDs. Every patient discharged with undifferentiated abdominal pain should be given clear instructions as to when it is necessary to return to the ED. If urolithiasis is excluded by means of a helical CT scan for a typical patient with flank pain, it is recommended that a contrast-enhanced CT scan be performed. Assay of LDH is useful when renal infarction is suspected. The optimal treatment of renal infarction is still uncertain, and the diagnosis is often late. The main focus is on preventing further damage to the patient.

## Consent

Written informed consent was obtained from the patient for the publication of this case report and any accompanying images. A copy of the written consent is available for review from the Editor-in-Chief of this journal.

## Abbreviations

ASA: acetylsalicylic acid
BP: blood pressure
CRP: C-reactive protein
CT: computed tomography
ECG: electrocardiogram
ED: emergency department
FMD: fibromuscular dysplasia
GFR: glomerular filtration rate
LDH: lactate dehydrogenase
MRA: magnetic resonance angiography
WBC: white blood cell
